# Efficient In Vitro Sterilization and Propagation from Stem Segment Explants of *Cnidoscolus aconitifolius* (Mill.) I.M. Johnst, a Multipurpose Woody Plant

**DOI:** 10.3390/plants11151937

**Published:** 2022-07-26

**Authors:** Min Gu, Youli Li, Huier Jiang, Shihu Zhang, Qingmin Que, Xiaoyang Chen, Wei Zhou

**Affiliations:** 1 State Key Laboratory for Conservation and Utilization of Subtropical Agro-Bioresources, South China Agricultural University, Guangzhou 510642, China; gumin08@163.com (M.G.); 2114965524@stu.scau.edu.cn (Y.L.); jianghuier2022@126.com (H.J.); shihu_zhang0229@163.com (S.Z.); qmque@scau.edu.cn (Q.Q.); 2 Guangdong Key Laboratory for Innovative Development and Utilization of Forest Plant Germplasm, Guangzhou 510642, China; 3 Guangdong Province Research Center of Woody Forage Engineering Technology, Guangzhou 510642, China; 4 College of Forestry and Landscape Architecture, South China Agricultural University, Guangzhou 510642, China

**Keywords:** *Cnidoscolus aconitifolius*, aseptic system, plant growth regulators, tissue culture

## Abstract

*Cnidoscolus aconitifolius* (Mill.) I.M. Johnst is a multipurpose woody plant. In this study, an in vitro efficient propagation system of stem segment explants derived from field-grown *C. aconitifolius* plants was established for the first time. The sterilization effect, axillary bud initiation, and proliferation efficiency of stem segments were evaluated. The results showed that the sterilization time of 0.1% mercuric chloride, the concentration of Plant Preservative Mixture (PPM), the pretreatment method, and the sampling season had significant effects on the sterilization of stem segments (*p* < 0.05). The type of medium and plant growth regulators (PGRs) affected the initiation of axillary buds, and the proliferation efficiency was significantly affected by PGRs. The results showed that the best sterilization method for stem segment explants was as follows: a pretreatment by rinsing with running water for 120 min, soaking in 75% ethanol for 50 s, soaking in 0.1% mercuric chloride for 10 min, and medium supplemented with 3 mL/L PPM. When inoculated on the medium in spring, the contamination rate was as low as 25.56%. The optimal initiation medium for axillary buds in stem segments was half-strength Murashige and Skoog (1/2 MS) medium supplemented with 0.5 mg/L 6-benzyladenine (6-BA). The induction rate was as high as 93.33%, and the mean length of axillary buds was 2.47 cm. The optimal proliferation medium was 1/2 MS medium supplemented with 4.0 mg/L 6-BA and 0.2 mg/L indole-3-butyric acid (IBA). The induction rate was up to 80.00%, the total proliferation coefficient was 4.56, and the net proliferation coefficient was 5.69. The 1/2 MS medium supplemented with 0.1 mg/L 6-BA and 1.5 mg/L indole-3-acetic acid (IAA) was most conducive to the elongation of the adventitious shoot, and the adventitious shoot of approximately 1 cm reached 1.93 cm after culturing for 14 days. The best medium for adventitious shoot rooting was 1/2 MS medium supplemented with 0.1 mg/L α-naphthalene acetic acid (NAA), the highest rooting rate was 82.00%, and the survival rate of transplanting was over 90%.

## 1. Introduction

*Cnidoscolus aconitifolius* also known as tree spinach is a hermaphroditic shrub of the Euphorbiaceae family with the characteristics of short flower lifespans, insect pollination, rapid growth, and a high resistance to adverse environmental conditions. It is native to the Yucatan peninsula of Mexico in Central America and is now commonly found in tropical and subtropical regions [1,2]. *C. aconitifolius* is an edible vegetable crop that tastes like spinach [3] but has a higher nutritional value [4]. It is not only rich in protein and vitamins but also contains a variety of amino acids and mineral elements necessary for the human body [5,6,7]. *C. aconitifolius* is also rich in medicinal properties. The hydroalcoholic extracts of *C. aconitifolius* leaves are rich in phenolic chemicals [8,9], which make it an excellent source of natural antioxidants. Adeniran et al. [10] showed that the ethanolic extracts of its roots, stems, and leaves had obvious inhibitory effects on *Bacillus subtilis* and *Escherichia coli*, and the antibacterial effects of all extracts were better than those of some common commercial antibiotics, such as tetracycline. *C. aconitifolius* extracts also showed the potential to lower blood sugar, protect the stomach, prevent precancerous lesions of the colon in rats, and protect the liver [11,12,13,14]. In addition, the addition of *C. aconitifolius* pellets to conventional feeds can significantly increase the nutrient content and crude protein digestibility of feeds [15,16,17], which can help to solve the world feed resource shortage problem. Moreover, it has been shown that the feeding of *C. aconitifolius* leaves to broiler chickens resulted in lower total cholesterol levels [18]. Thus, *C. aconitifolius* is a multipurpose woody plant that is receiving increasing attention worldwide.

Seeds of cultivated *C. aconitifolius* are rare [19,20], and most of them are propagated by cuttings. Tissue culture has the advantages of a high efficiency, a high reproduction coefficient, and the ability to maintain excellent woody traits. In addition, the tissue culture of *C. aconitifolius* has the potential to promote the production of medicinal secondary metabolites [21,22]. Therefore, to ensure its large-scale production and utilization, research on *C. aconitifolius* in vitro regeneration in tissue culture is necessary.

Tissue culture mainly refers to the proliferation and growth of tissues or organs in a sterile and controlled environment. It is not only essential for basic research but also has commercial application value [23]. Plant tissue culture is usually used as an in vitro biotechnology tool for the clonal propagation of plants with desirable traits [24,25], the investigation of plant development process [26], obtaining plant materials without virus [27], the establishment of genetic transformation system [28,29], and the breeding of plant polyploids.

To the best of our knowledge, there have been no previous reports on the in vitro propagation of *C. aconitifolius* in tissue culture. This study is the first to establish an in vitro tissue culture propagation system using stem segments derived from field-grown biennial *C. aconitifolius* plants as explants, providing a new propagation method for *C. aconitifolius*. The establishment of a sterile system is the precondition for establishing a plant regeneration system. Biennial *C. aconitifolius* can be used to screen out excellent individual plants. Therefore, the study of stem segment explants of field-grown biennial *C. aconitifolius* can be used for the propagation of excellent individual plants, which can maintain the excellent traits of the parent. However, with the extension of field-planting time, the gradual increase of endophytes in *C. aconitifolius* makes it difficult to sterilize, and the rich phenolic substances in *C. aconitifolius* further increase the difficulty of sterilization [30]. Therefore, this paper studied the effects of mercury chloride treatment time, antibiotics, pretreatment methods, and sampling time on the sterilization of stem segment explants of field-grown *C. aconitifolius* plants and explored the effect of medium and plant growth regulators (PGRs) on the propagation efficiency of explants. These data were successfully used to establish a high-efficiency tissue culture propagation system of *C. aconitifolius* for the first time, which ensured the efficient regeneration of *C. aconitifolius*, promoted its large-scale production and utilization, and laid a foundation for future research on polyploid breeding and genetic transformation.

## 2. Materials and Methods

### 2.1. Plant Material Preparation and Growth Conditions

The plant materials used in this study were all collected from the nursery of South China Agricultural University and young stem segments with bud points were selected as explants from field-grown biennial *C. aconitifolius*, from Texas and Florida, USA.

Unless otherwise specified, all media in this experiment contained 0.6% agar and 3% sucrose, with a pH of 5.8–6.2. All sterilization experiments and axillary bud induction experiments were cultured in the dark for 2 days after inoculation and then placed under the conditions of 12 h of light per day, the light intensity was 2100–2500 lx, and the culture temperature was 23–25 °C. For the proliferation culture test, the axillary buds were directly placed under the conditions of 12 h of light per day, the light intensity was 2100–2500 lx, and the culture temperature was 23–25 °C.

### 2.2. Sterilization of Explants

#### 2.2.1. Examining the Effect of Different Sterilization Times of 0.1% Mercuric Chloride

In this experiment, young stem segments with bud points were used as explants. The explants were cleaned of dust and other debris in a detergent solution and then rinsed for 120 min in slow running water to remove residues from the explant surface. Stem segments were sterilized on an ultraclean bench as follows: sterilized with 75% ethanol for 50 s, washed with sterile water, further sterilized with 0.1% mercuric chloride for 5, 8, 10, 12, and 15 min, and then washed with sterile water 5 times. Finally, the stem segments were vertically inoculated into a hormone-free Murashige and Skoog (MS) medium. The treatments were repeated three times, with each replicate consisting of 30 explants. After 14 days of culture, the contamination rate, death rate, and survival rate were recorded.

#### 2.2.2. Examining the Effect of Different Concentrations of the Antibiotic PPM on Explant Sterilization

Stem segments were rinsed with running water for 120 min for pretreatment, sterilized with 75% ethanol for 50 s, further sterilized with 0.1% mercuric chloride for 10 min, and then washed with sterile water five times. Finally, the sterilized stem segment explants were vertically inoculated into MS medium supplemented with various concentrations of Plant Preservation Mixture (PPM) (0, 1, 2, 3, and 4 mL/L) and 3% sucrose. The treatments were repeated three times, with each replicate consisting of 30 explants. After 14 days of culture, the contamination rate, death rate, and survival rate were recorded.

#### 2.2.3. Examining the Effect of Different Pretreatment Methods on Explant Sterilization

Stem segment explants were pretreated by two pretreatment methods: running water rinsing (0, 30, 60, and 120 min) and 0.1% carbendazim solution soaking (5, 10, 15, 20, and 30 min), and then the stem segments were treated with the corresponding optimal sterilization method. After sterilization, the stem segment explants were vertically inoculated into MS medium supplemented with 3 mL/L PPM and 3% sucrose. The treatments were repeated three times, with each replicate consisting of 30 explants. After 14 days of culture, the contamination rate, death rate, and survival rate were recorded.

#### 2.2.4. Examining the Effect of Sampling Time on Explant Sterilization

The explants were sampled in March–May, June–August, September–November, and December–February. After the explants were treated with the corresponding optimal pretreatment and sterilization method, they were vertically inoculated in MS medium supplemented with 3 mL/L PPM and 3% sucrose. The treatments were repeated three times, with each replicate consisting of 30 explants. After 14 days of culture, the contamination rate, death rate, and survival rate were recorded.

### 2.3. Induction of Axillary Buds in Stem Segments

Stem segments with similar growth status were selected and treated with the corresponding optimal pretreatment and sterilization method, and then they were vertically inoculated into MS, half-strength Murashige and Skoog (1/2 MS), and B5 medium supplemented with various concentrations of 6-benzyladenine (6-BA) (0, 0.5, and 1.0 mg/L), 3 mL/L PPM, and 3% sucrose. There were 9 treatments in total, and the treatments were repeated three times, with each replicate consisting of 30 explants. After 14 days of culture, the contamination rate, death rate and the survival rate were recorded.

### 2.4. Induction of Adventitious Shoot

Axillary buds longer than 1.5 cm were cut with a sterile scalpel from stem segments and then vertically inoculated into the 1/2 MS medium supplemented with various concentrations of indole-3-butyric acid (IBA) (0.1, 0.2, and 0.5 mg/L), 6-BA (1, 2, 4, and 5 mg/L), and 3% sucrose. There were 12 treatments in total, and the treatments were repeated three times, with each replicate consisting of 30 explants. After 30 days, the induction rate and proliferation coefficient of adventitious shoots were calculated.

### 2.5. Induction of Adventitious Shoot Elongation

Adventitious shoots approximately 1 cm in length were cut from the clump shoots with a sterile scalpel and then vertically inoculated into 1/2 MS medium supplemented with various concentrations of 6-BA (0, 0.1, and 0.2 mg/L), indole-3-acetic acid (IAA) (1.0, 1.5, and 2.0 mg/L), and 3% sucrose. Elongation culture before rooting was performed. There were 9 treatments in total, and the treatments were repeated three times, with each replicate consisting of 30 explants. After 14 days, the length of the adventitious shoots was calculated.

### 2.6. Induction of Adventitious Shoot Rooting

Regenerated adventitious shoots longer than 2 cm were selected and vertically inoculated into the 1/2 MS rooting medium supplemented with various concentrations of α-naphthalene acetic acid (NAA) (0, 0.1, 0.2, and 0.5 mg/L). The treatments were repeated three times, with each replicate consisting of 30 explants. After 14 days, the rooting rate of adventitious shoots was calculated.

### 2.7. Plant Domestication and Transplanting

Healthy, regenerated plantlets with well-developed roots were individually removed from the tissue culture room and cultured under natural light for 5–7 days. Then, the rooted plants were removed from their tissue culture bottles, and the agar was rinsed away under running tap water. The plantlets were transferred to a sterilized matrix containing a mixture of perlite and peat soil (1:1) covered with a polythene film, and the seedlings were sprayed with water daily to maintain humidity. After 2–3 weeks of acclimation, the regenerated seedlings were transplanted to a field for further cultivation. Plantlets were deemed to have been successfully transplanted if they grew new terminal buds.

### 2.8. Statistical Analyses

In the sterilization experiments, the contamination rate, death rate, and survival rate were recorded and calculated by dividing the number of contaminated explants, the number of uncontaminated but dead explants, and the number of uncontaminated and surviving explants by the number of inoculations×100. In the axillary bud induction experiments, the frequency of axillary bud induction was calculated by dividing the number of stem segments that induced axillary buds by the number of inoculated stem segments*100. In the adventitious shoot induction experiments, the frequency of adventitious shoot induction was calculated by dividing the number of explants that induced adventitious shoots by the number of inoculated explants*100, and the total proliferation coefficient was calculated by dividing the total number of adventitious shoots by the total number of explants, and the net proliferation coefficient was calculated by dividing the total number of adventitious shoots by the number of explants that induced adventitious shoots. In the adventitious shoot elongation experiments, the shoot length of each shoot was recorded. In the rooting experiments, the rooting induction rate was calculated by dividing the number of adventitious shoots by the number of adventitious shoots*100.

Statistical analyses were performed using SPSS software (version 19.0; SPSS Inc., Chicago, IL, USA). Means were compared using Duncan’s multiple range test or the L.S.D. test, with a significance level of 0.05. Graphs were produced using SPSS (version 19.0) and Microsoft Office Excel (Microsoft Corp., Redmond, WA, USA)

## 3. Results

### 3.1. Effect of 0.1% Mercuric Chloride with Different Soaking Times on Explant Sterilization

**Figure 1:**
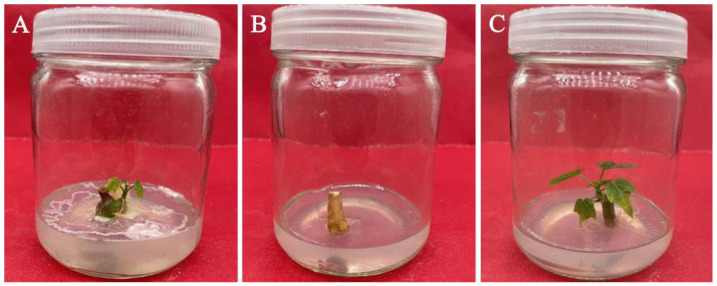
Performance of sterilization on *C. aconitifolius* stem segment explants. (**A**) Contaminated stem segment explants; (**B**) uncontaminated but dead stem segment explants; and (**C**) uncontaminated and surviving stem segment explants.

It was observed that the contamination rate of stem segments sterilized in 0.1% mercuric chloride for 15 min was the lowest (48.89%), followed by the sterilization of 12 min (52.22%), and the contamination rate of stem segments sterilized in 0.1% mercuric chloride for 10 min was not significantly different from that of the sterilization for 12 min or 15 min (*p* < 0.05) (Table 1, Figure 1). The contamination rate was still higher when sterilized in 0.1% mercuric chloride for 5 min or 8 min. Mercury chloride has a certain toxic effect on plants, and this effect tends to be more pronounced with increasing treatment time [31]. The death rate reached the highest value (8.89%) when sterilized with 0.1% mercuric chloride for 15 min, which was significantly different from that of sterilization for 5 min, 8 min, and 10 min, but not significantly different from that of sterilization for 12 min. Therefore, the sterilization with 0.1% mercuric chloride for 10 min had the highest survival rate (44.44%) due to a lower death rate and a lower contamination rate.

### 3.2. Effect of Different Concentrations of the Antibiotic PPM on Explant Sterilization

The results showed that when the sterilization time of 0.1% mercuric chloride on *C. aconitifolius* stem segment explants was fixed, the addition of PPM to the medium could significantly reduce the contamination rate of explants. The contamination rate decreased with the increase in the antibiotic PPM concentration, but the decreasing gradient gradually decreased, and the lowest value was recorded when PPM was 4 mL/L (26.67%) (Table 2). PPM is a broad-spectrum antibiotic, and a suitable concentration of PPM can effectively inhibit contamination in plant tissue culture and promote the development of explants. From the data, there was no significant differences in the contamination rate and the survival rate of stem segment explants when the PPM concentrations were 3 mL/L and 4 mL/L (*p* < 0.05). To reduce the risk of inhibition by PPM as much as possible, 3 mL/L PPM was added to the medium during subsequent experiments.

### 3.3. Effect of Different Pretreatment Methods on Explant Sterilization

Two pretreatment methods were used for the stem segments of *C. aconitifolius*: rinsing with running water and soaking with 0.1% carbendazim. The results showed that both pretreatment methods could significantly reduce the contamination rate (*p* < 0.05) (Table 3). When the stem segments were rinsed with running water for 150 min, the contamination rate reached the lowest value (24.44%), and the survival rate was the highest (73.33%), which was not significantly different from that rinsing for 120 min but was significantly different from other treatments (*p* < 0.05). Rinsing with running water can effectively remove harmful microorganisms on the stem segments’ surface. The 0.1% carbendazim soaking could also effectively reduce the contamination rate, but at the same time, there was a risk of increasing the death rate, and it caused great damage to the stem segment explants of *C. aconitifolius*. When the 0.1% carbendazim soaking time reached 30 min, the death rate (10.00%) was significantly higher than that of other treatments (*p* < 0.05), and the contamination rate (38.89%) was also significantly higher than that of running water rinsing for 120 min (*p* < 0.05). To ensure the sterilization effect while saving time, the best pretreatment method was to rinse with running water for 120 min.

### 3.4. Effect of Sampling Season on Explant Sterilization

The contamination rate of *C. aconitifolius* stem segments reached the lowest (25.56%) in March–May and reached the highest (32.22%) in December–February (Table 4). There was a significant difference between spring and winter (*p* < 0.05), which may be related to the growth habit of *C. aconitifolius*. Winter was the time of year when *C. aconitifolius* was less active, the metabolic activity was slow, and the endophytes were easy to breed, which led to an increase in the contamination rate. Due to the low temperature in winter, it was not suitable for survival, and the stem segments of *C. aconitifolius* were in a poor physiological state, the death rate was also higher than that in other seasons. Correspondingly, the highest survival rate (73.33%) of stem segments (73.33%) appeared in March–May, and the lowest (64.44%) in December–February, the difference was significant (*p* < 0.05).

### 3.5. Effect of Medium Types and PGRs on Axillary Bud Induction

**Figure 2:**
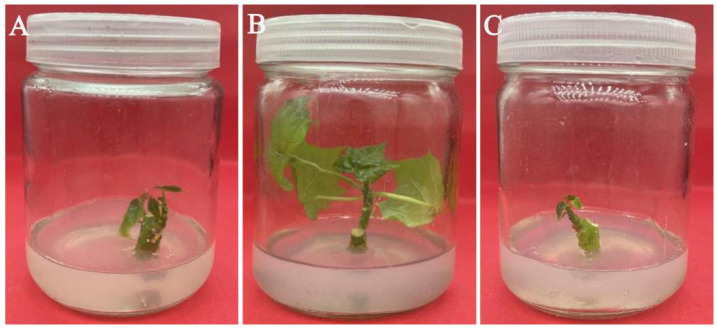
Axillary bud induction of stem segments on different media. (**A**) MS medium supplemented with 0.5 mg/L 6-BA; (**B**) 1/2 MS medium supplemented with 0.5 mg/L 6-BA; and (**C**) B5 medium supplemented with 0.5 mg/L 6-BA.

A total of nine different treatments were set up for axillary bud induction of stem segments. The purpose of adding cytokinin to the medium was mainly to promote cell division and differentiate adventitious shoots from calli or organs. Both the medium and 6-BA had significant effects on the frequency of axillary bud induction and mean length of axillary buds, and their interaction also had a significant effect on the mean length of axillary buds, but the effect on the frequency of axillary bud induction was not significant (*p* < 0.05) (Table 5, Figure 2). Among the three different media, the length of axillary buds in the treatments with 1.0 mg/L 6-BA was always significantly shorter than those in the other two concentrations, and the length of axillary buds in the treatments with 0.5 mg/L 6-BA was always longer than those supplemented with 0 mg/L 6-BA (*p* < 0.05) (Table 6). Regardless of the 6-BA concentration, the mean length of axillary buds in 1/2 MS medium was always longer than that in MS medium or B5 medium, but only when 6-BA was 0.5 mg/L, the difference was significant, the frequency of axillary bud induction also reached the maximum value (93.33%) (*p* < 0.05). In summary, the optimal medium for the induction of axillary buds in the stem segments of *C. aconitifolius* was 1/2 MS medium supplemented with 0.5 mg/L 6-BA.

### 3.6. Effects of PGRs on Adventitious Shoot Induction and Proliferation

**Figure 3:**
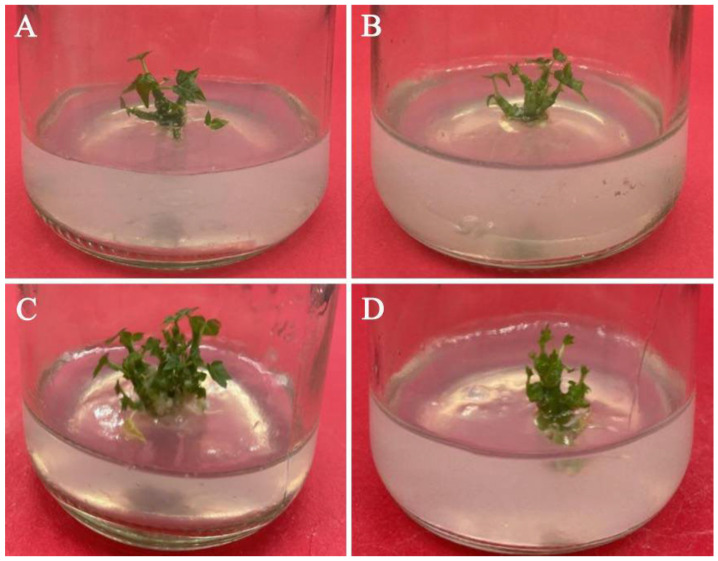
Proliferation and induction of adventitious shoot on medium with different 6-BA concentrations: (**A**) 1/2 MS medium supplemented with 0.2 mg/L IBA and 1.0 mg/L 6-BA; (**B**) 1/2 MS medium supplemented with 0.2 mg/L IBA and 2.0 mg/L 6-BA; (**C**) 1/2 MS medium supplemented with 0.2 mg/L IBA and 4.0 mg/L 6-BA; and (**D**) 1/2 MS medium supplemented with 0.2 mg/L IBA and 5.0 mg/L 6-BA.

Axillary buds, approximately 2 cm long, were stripped from their bases and transferred to the proliferation medium. 6-BA, IBA, and their interaction all had significant effects on the frequency of shoot induction, net proliferation coefficient, and total proliferation coefficient (*p* < 0.05) (Table 7, Figure 3). On the one hand, regardless of the 6-BA concentration, the frequency of shoot induction, net proliferation coefficient, and total proliferation coefficient in the treatment containing 0.1 mg/L IBA were always lower than that containing 0.2 mg/L IBA or 0.5 mg/L IBA. However, when the concentration of 6-BA was 5.0 mg/L, the net proliferation coefficient of the treatment containing 0.1 mg/L IBA was not significantly different from that containing 0.2 mg/L IBA, and the promoting effect of the treatments containing 0.1 mg/L IBA on the proliferation of adventitious shoots were always significantly weaker than those containing 0.2 mg/L IBA (*p* < 0.05) (Table 8). On the other hand, no matter what the IBA concentration was, the proliferation effect of the treatment containing 4.0 mg/L 6-BA was always better than the other three concentrations. However, when the IBA concentration was 0.1 mg/L, the net proliferation coefficient of the treatment containing 4.0 mg/L 6-BA was not significantly different from that containing 5.0 mg/L 6-BA, and the proliferation effect of the treatment containing 4.0 mg/L 6-BA was always significantly different from the other three concentrations (*p* < 0.05). In summary, the highest induction rate (80.00%), the largest total proliferation coefficient (4.56), and the largest net proliferation coefficient (5.69) were all recorded when the concentration of 6-BA was 4.0 mg/L and the concentration of IBA was 0.2 mg/L.

### 3.7. Effect of PGRs on Adventitious Shoot Elongation

**Figure 4:**
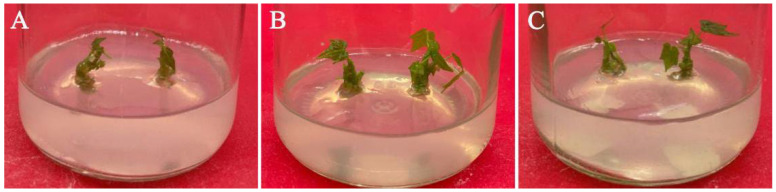
Elongation of adventitious buds on medium with different IAA concentrations: (**A**) 1/2 MS medium supplemented with 0.1 mg/L 6-BA and 1.0 mg/L IAA; (**B**) 1/2 MS medium supplemented with 0.1 mg/L 6-BA and 1.5 mg/L IAA; and (**C**) 1/2 MS medium supplemented with 0.1 mg/L 6-BA and 2.0 mg/L IAA.

Based on the adventitious shoot proliferation results, the effect of the combination of low concentrations of 6-BA and different concentrations of IAA on shoot elongation was also investigated. Approximately 1 cm of adventitious shoots were cut from the clump shoots and transferred to the elongation medium, and 6-BA concentrations (0, 0.1, and 0.2 mg/L) and IAA concentrations (1.0, 1.5, and 2.0 mg/L) were added for elongation culture before rooting. The results showed that 6-BA, IAA, and their interactions all had significant effects on the elongation of the adventitious shoot, among which IAA had the greatest effect (*p* < 0.05) (Table 9, Figure 4); each treatment could promote the elongation of the adventitious shoots and had a certain effect of strengthening seedlings. Regardless of the concentration of 6-BA, the length of adventitious shoots of the treatment with 1.0 mg/L IAA was always significantly shorter than that supplemented with 1.5 mg/L IAA or 2.0 mg/L IAA (*p* < 0.05) (Table 10). When IAA was 1.0 mg/L, the concentration of 6-BA had no significant effect on the length of adventitious shoots. However, when the concentration of IAA was 1.5 mg/L or 2.0 mg/L, the length of adventitious shoots in the treatment with 0.1 mg/L 6-BA was significantly longer than that supplemented with 0 mg/L 6-BA (*p* < 0.05). The maximum value (1.93 cm) in all treatments was recorded when the concentration of 6-BA was 0.1 mg/L and the concentration of IAA was 1.5 mg/L (*p* < 0.05).

### 3.8. Adventitious Shoot Rooting

**Figure 5:**
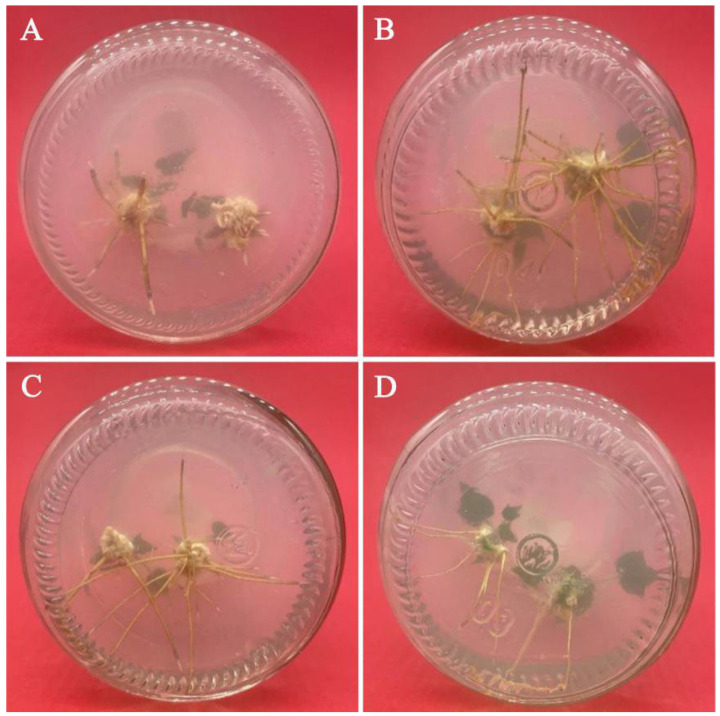
Rooting of adventitious shoots on medium with different NAA concentrations: (**A**) 1/2 MS medium supplemented with 0 mg/L NAA; (**B**) 1/2 MS medium supplemented with 0.1 mg/L NAA; (**C**) 1/2 MS medium supplemented with 0.2 mg/L NAA; and (**D**) 1/2 MS medium supplemented with 0.5 mg/L NAA.

Adventitious shoots about 1.5–2.0 cm in length were transferred to a rooting medium with different concentrations of NAA (0, 0.1, 0.2, and 0.5 mg/L). Although adventitious shoots under all NAA conditions were rooted, the concentration of NAA had a significant effect on the induction efficiency of adventitious shoot rooting (*p* < 0.05) (Table 11, Figure 5). When the concentration of NAA was 0 mg/L, the rooting rate of the adventitious shoots of *C. aconitifolius* was only 42.22%, the adventitious roots were short, and the number of the roots was relatively small. However, when the concentration of NAA was 0.1–0.2 mg/L, the rooting rate of the adventitious shoots was >70.00%, the mean number of roots was >4.00, and the mean length of the roots was >3.00 cm. At 0.1 mg/L NAA, the rooting rate reached the highest value (82.00%), the mean number of roots was up to 4.84, and the mean length of root was up to 3.91 cm.

### 3.9. Plant Domestication and Transplanting

To test the transplanting survival rate of *C. aconitifolius* plants cultured from stem segment explants, explants with root length of 3–5 cm were transplanted into pots under the soil and natural light conditions and then transplanted to the field after 2–3 weeks of acclimation. The tissue culture seedlings were transplanted and survived after the growth of new terminal buds, and the transplanted survival rate was over 90%.

## 4. Discussion

Efficient propagation is an important guarantee for the large-scale production and utilization of *C. aconitifolius*. To the best of our knowledge, this study is the first to conduct in vitro sterilization experiments using stem segments derived from field-grown *C. aconitifolius* plants. The experiment material was not only easy to obtain but could also maintain the excellent properties of the parent. A solution of 0.1% mercuric chloride was used for the sterilization of field grown *C. aconitifolius* stem segments. Mercury chloride is a commonly used effective surface fungicide for explant sterilization [32]. Rafiq et al. [33] showed that the scale of Oriental Hybrid *Lilium* cv. Ravenna was sterilized with 0.1% mercuric chloride for 10 min, and the culture asepsis rate reached 77.08%. Poobathy et al. [34] found that surface sterilization of *Ludisia discolor* with mercuric chloride resulted in a contamination rate as low as 7.70%. However, judging from the data, the contamination rate of *C. aconitifolius* was still high after mercury chloride treatment. The reason for the analysis may be that there are many endophytes in the stem segments of field-grown *C. aconitifolius*, and the effect of surface sterilization is limited.

When the endophytes in the explants are more serious and sterilants such as mercuric chloride can not control contamination in plant tissue culture, antibiotics can be added to the medium for inhibition. The results showed that PPM could effectively reduce the contamination rate of *C. aconitifolius* stem segments. Li et al. [35] also found that adding 25 mg/L PPM to the medium did not cause contamination even without sterilization with 75% ethanol. In the propagation of *Panicum virgatum* L., the addition of 0.2% PPM to the medium after surface sterilization resulted in a significant reduction in bacterial and fungal contamination [36]. However, if the concentration of PPM is too high, it will also cause adverse effects on the development of explants and even lead to explant death [37,38,39]. In tissue culture experiments of anthurium and cauliflower, appropriate concentrations of PPM increased the number of axillary bud germinations [40,41]. When 6–10 mL/L PPM was added to the medium of *Petunia* *hybrida*, the number of newly germinated leaves was significantly lower than that in the medium supplemented with 3–5 mL/L PPM [42]. Therefore, the appropriate concentration of PPM should be explored in the experiment.

Plants growing in the field contain many bacteria. If the collected explants are directly sterilized on a sterile operating table, sterilization is difficult. The results of the study further showed that rinsing with running water could remove contaminants on the surface of explants, and carbendazim was also a common effective antibacterial agent [43]. Studies have shown that different explant sampling seasons can also affect the sterilization effect [44], which is consistent with our findings, mainly due to differences in plant growth activities in different seasons.

The results showed that the concentration of 6-BA had a greater effect on the induction of axillary buds than the type of medium. An appropriate concentration of 6-BA contributes to the elongation of axillary buds, and 1.0 mg/L 6-BA was too high for the growth of *C. aconitifolius* axillary buds. Ho et al. [45] also found that 6-BA promoted axillary bud induction in nodal sections of *Acacia confusa*. When the 6-BA concentration was 0.5 mg/L, 1/2 MS medium was the optimal medium, and the mean length of axillary buds reached the maximum (2.47 cm). Appropriate 6-BA concentration could maximize the effect of 1/2 MS medium on the growth of axillary buds. From the data, the optimal concentration of inorganic salts for the induction of axillary buds in *C. aconitifolius* stem segment explants was lower. High concentrations of inorganic salts inhibited the development and induction of axillary buds, which may be the reason for the poor length of axillary buds in MS and B5 media. The effect of the medium on the regeneration efficiency of explants has also been reported in other species. A study by Zahid et al. [46] showed that the medium had a significant effect on the regeneration efficiency of rhizome explants of *Zingiber officinale* Roscoe. The high concentration of inorganic salts such as nitrogen and potassium contained in MS medium can meet the nutrient conditions required for rapid growth and promote the growth and long-term survival of the culture, while B5 medium contains low concentrations of ammonium and high concentrations of nitrate, thiamine hydrochloride [47].

Cytokinins normally interact with auxins to promote the proliferation of adventitious shoots. The adventitious shoot proliferation culture of *C. aconitifolius* showed that 6-BA, IBA, and their interaction had significant effects on the proliferation of adventitious shoots. When IBA concentration was 0.1 mg/L, the proliferation effect of 6-BA on the adventitious shoots was weakened, so an appropriate increase in the concentration of IBA could effectively promote the proliferation of adventitious shoots. The treatments supplemented with 4.0 mg/L 6-BA had a better proliferation effect than those supplemented with 1.0 mg/L 6-BA or 2.0 mg/L 6-BA, indicating that *C. aconitifolius* axillary bud explants were not sensitive to 6-BA. Moreover, when the concentration of 6-BA was 5.0 mg/L, the frequency of shoot induction and proliferation coefficient of adventitious shoots were always lower than those of 4.0 mg/L 6-BA, the induced adventitious shoots generally could not grow normally, and the abnormal rate of adventitious shoots was high, indicating that the 5.0 mg/L 6-BA was too high. The results showed that the optimal concentrations of adventitious shoot proliferation were 4.0 mg/L 6-BA and 0.2 mg/L IBA. Under these suitable concentrations, the axillary bud explants produced abundant adventitious shoots and the adventitious shoots grew better. Furthermore, many effective adventitious shoots could be used for elongation and rooting, and the utilization rate was high in the later stage. Studies on other species also showed that the combination of 6-BA and IBA was beneficial to proliferation; in the propagation of *Securidaca longipedunculata* (Fresen) on MS proliferation medium supplemented with 1.5 mg/L 6-BA and 0.1 mg/L IBA, the mean number of explant shoots was up to 8.5 [48]. To make full use of the adventitious shoot, elongation culture was carried out. The results showed that the effect of 6-BA concentration on the length of the adventitious shoots was not as obvious as that of IAA, but still significant, which was consistent with the results of Hou et al. [25] on *Sapium sebiferum* Roxb. From the results, IAA was easily decomposed when exposed to light, and when the concentration of IAA was 1.0 mg/L, the effect of 6-BA on the elongation of the adventitious shoots of *C. aconitifolius* was relatively small. However, when the concentration of IAA was appropriately increased, the difference in the length of adventitious shoots between treatment containing 6-BA and the treatment without 6-BA became larger. The combination of a low concentration of 6-BA and an appropriate concentration of IAA can effectively promote the elongation of the adventitious shoots and ensure that more adventitious shoots can be used for rooting.

According to Mao et al. [49], 1/2 MS with 0.1 mg/L NAA was beneficial for the rooting of the adventitious shoots of *Toona* *ciliata*. The results of adventitious shoot rooting of *C. aconitifolius* also showed that NAA was beneficial to promoting the rooting of adventitious shoots, the rooting percentage, mean number of roots, and mean length of roots of the adventitious shoots first increased and then decreased with increasing NAA concentration, which was consistent with the results of the rooting study of *Cannabis sativa* by Ioannidis et al. [50]. The observation of adventitious root morphology showed that a strong root system was not easy to break during seedling raising and transplanting, which was beneficial to the transplanting survival rate.

## 5. Conclusions

The effects of 0.1% mercuric chloride soaking time, PPM concentration, pretreatment method, and sampling season on the sterilization of stem segment explants of *C. aconitifolius* were studied. Overcoming the problem of poor sterilization effect of field-grown *C. aconitifolius* caused by prolonged field-planting time and abundant phenolic substances in plants found in previous preliminary experiments, the aseptic system of stem segments of field-grown biennial *C. aconitifolius* was established, which is conducive to the efficient propagation of excellent individual plants. The successful establishment of the aseptic system is the basis for propagation in vitro, which provides a guarantee for the tissue culture of *C. aconitifolius* [30]. Based on establishing the aseptic system, the factors affecting the initiation of axillary bud, the proliferation, elongation, and rooting of the adventitious shoots were further studied. It was shown that the type of medium and PGRs concentration influenced the induction and the growth of axillary bud in stem segments, and the appropriate type and concentration of PGRs were beneficial to the proliferation, elongation, and the rooting of adventitious shoots.

## Figures and Tables

**Table 1:** Effect of 0.1% mercuric chloride on explant sterilization.

0.1% Mercuric Chloride (min)	Contamination (%)	Death (%)	Survival (%)
5	74.44 ± 5.09 a	0.00 ± 0.00 b	25.56 ± 5.09 c
8	65.56 ± 1.93 b	0.00 ± 0.00 b	34.44 ± 1.93 b
10	54.44 ± 3.85 c	0.00 ± 1.92 b	44.44 ± 5.09 a
12	52.22 ± 5.09 c	6.67 ± 0.00 a	41.11 ± 5.09 a
15	48.89 ± 5.09 c	8.89 ± 3.85 a	42.22 ± 1.92 a

MS medium supplemented with 3% sucrose. Values are the mean ± SD of three replicates, each with 30 explants. Means with the same letters in the same columns are not significantly different from each other at *p* < 0.05 (according to Duncan’s multiple range test).

**Table 2:** Effect of PPM on explant sterilization.

PPM (ml/L)	Contamination (%)	Death (%)	Survival (%)
0	55.55 ± 3.85 a	1.11 ± 1.92 a	43.33 ± 3.34 d
1	44.44 ± 1.93 b	0.00 ± 0.00 a	55.56 ± 1.93 c
2	36.67 ± 3.34 c	0.00 ± 0.00 a	63.33 ± 3.34 b
3	28.89 ± 3.85 d	1.11 ± 1.92 a	70.00 ± 3.34 a
4	26.67 ± 0.00 d	1.11 ± 1.92 a	72.22 ± 1.93 a

MS medium supplemented with 3% sucrose. Values are the mean ± SD of three replicates, each with 30 explants. Means with the same letters in the same columns are not significantly different from each other at *p* < 0.05 (according to Duncan’s multiple range test).

**Table 3:** Effect of different pretreatment methods on explant sterilization.

Pretreatment Methods	Contamination (%)	Death (%)	Survival (%)
Running water rinsing for 0 min	52.22 ± 1.92 a	0.00 ± 0.00 b	47.78 ± 1.92 e
Running water rinsing for 30 min	45.56 ± 1.92 b	0.00 ± 0.00 b	54.44 ± 1.92 cd
Running water rinsing for 60 min	38.89 ± 1.92 c	0.00 ± 0.00 b	61.11 ± 1.92 b
Running water rinsing for 120 min	26.67 ± 0.00 d	1.11 ± 1.92 b	72.22 ± 1.92 a
Running water rinsing for 150 min	24.44 ± 1.92 d	2.22 ± 1.92 b	73.33 ± 3.33 a
0.1% Carbendazim soaking for 5 min	41.11 ± 1.92 c	0.00 ± 0.00 b	58.89 ± 1.92 bc
0.1% Carbendazim soaking for 10 min	47.78 ± 1.92 b	0.00 ± 0.00 b	52.22 ± 1.92 de
0.1% Carbendazim soaking for 20 min	47.78 ± 1.92 b	1.11 ± 1.92 b	51.11 ± 1.92 de
0.1% Carbendazim soaking for 30 min	38.89 ± 1.92 c	10.00 ± 5.77 a	51.11 ± 6.94 de

MS medium supplemented with 3% sucrose. Values are the mean ± SD of three replicates, each with 30 explants. Means with the same letters in the same columns are not significantly different from each other at *p* < 0.05 (according to Duncan’s multiple range test).

**Table 4:** Effect of sampling season on explant sterilization.

Sampling Season	Contamination (%)	Death (%)	Survival (%)
March–May	25.56 ± 1.92 c	1.11 ± 1.92 a	73.33 ± 3.33 a
June–August	27.78 ± 1.92 bc	2.22 ± 1.92 a	70.00 ± 3.33 ab
September–November	31.11 ± 1.92 ab	1.11 ± 1.92 a	67.78 ± 3.85 ab
December–February	32.22 ± 1.92 a	3.33 ± 0.00 a	64.44 ± 1.92 b

MS medium supplemented with 3% sucrose. Values are the mean ± SD of three replicates, each with 30 explants. Means with the same letters in the same columns are not significantly different from each other at *p* < 0.05 (according to Duncan’s multiple range test).

**Table 5:** Effect of medium types and 6-BA on axillary bud induction.

Medium	6-BA (mg/L)	Frequency of Axillary Bud Induction (%)	Mean Length of Axillary Buds (cm)
MS	0	88.89 ± 1.92 c	1.56 ± 0.45 b
MS	0.5	92.22 ± 1.92 ab	1.62 ± 0.71 b
MS	1.0	88.89 ± 1.92 c	1.34 ± 0.39 c
1/2 MS	0	90.00 ± 0.00 bc	1.61 ± 0.38 b
1/2 MS	0.5	93.33 ± 0.00 a	2.47 ± 0.67 a
1/2 MS	1.0	87.78 ± 1.92 cd	1.35 ± 0.48 c
B5	0	88.89 ± 1.92 c	1.48 ± 0.33 bc
B5	0.5	88.89 ± 1.92 c	1.59 ± 0.39 b
B5	1.0	85.56 ± 1.92 d	1.33 ± 0.31 c
F value			
Medium		4.20 *	37.24 ***
6-BA		21.60 ***	82.17 ***
Medium × 6-BA		2.40 ^ns^	26.27 ***

Medium supplemented with 3% sucrose. Values are the mean ± SD of three replicates, each with 30 explants. Means with the same letters in the same columns are not significantly different from each other at *p* < 0.05 (according to Duncan’s multiple range test). F value represented * = *p* < 0.05, *** = *p* < 0.001, and ^ns^ = not significant.

**Table 6:** Effect of medium type and 6-BA on the mean length of axillary buds (cm).

	6-BA (mg/L)	MS	1/2 MS	B5
Medium				
0		1.56 ± 0.45 a (AB)	1.61 ± 0.38 b (A)	1.48 ± 0.33 a (A)
0.5		1.62 ± 0.71 a (B)	2.47 ± 0.67 a (A)	1.59 ± 0.39 a (B)
1.0		1.34 ± 0.39 b (A)	1.35 ±0.48 c (A)	1.33 ± 0.31 b (A)
F value				
Medium: 37.24 *** 6-BA: 82.17 *** Medium × 6-BA: 26.27 ***				

Medium supplemented with 3% sucrose. Values are the mean ± SD of three replicates, each with 30 explants. At the same medium type, means followed by the same lowercase letter in each column are not significantly different from each other at *p* < 0.05 (according to L.S.D. test). At the same 6-BA concentration, means followed by the same uppercase letter (in parenthesis) in each row are not significantly different from each other at *p* < 0.05 (according to L.S.D. test). F value represented *** = *p* < 0.001.

**Table 7:** Effects of 6-BA and IBA on adventitious shoot induction and proliferation.

6-BA (mg/L)	IBA (mg/L)	Frequency of Shoot Induction (%)	Net Proliferation Coefficient	Total Proliferation Coefficient
1.0	0.1	22.22 ± 3.85 f	2.70 ± 0.67 e	0.60 ± 1.18 d
1.0	0.2	42.22 ± 3.85 cd	3.42 ± 0.96 cde	1.44 ± 1.82 cd
1.0	0.5	31.11 ± 3.85 e	2.79 ± 0.80 e	0.87 ± 1.38 cd
2.0	0.1	28.89 ± 3.85 ef	3.00 ± 0.91 de	0.87 ± 1.45 cd
2.0	0.2	44.44 ± 3.85 c	3.90 ± 1.17 bc	1.73 ± 2.10 bc
2.0	0.5	35.56 ± 3.85 de	3.63 ± 0.96 bcd	1.29 ± 1.84 cd
4.0	0.1	42.22 ± 3.85 cd	4.00 ± 0.82 bc	1.69 ± 2.07 bc
4.0	0.2	80.00 ± 6.67 a	5.69 ± 1.45 a	4.56 ± 2.64 a
4.0	0.5	57.78 ± 3.85 b	4.35 ± 1.20 b	2.69 ± 2.35 b
5.0	0.1	28.89 ± 3.85 ef	3.46 ± 0.52 cde	0.87 ± 1.47 cd
5.0	0.2	44.44 ± 3.85 c	3.85 ± 0.75 bc	1.73 ± 1.94 bc
5.0	0.5	35.56 ± 3.85 de	3.69 ± 0.79 bcd	1.20 ± 1.78 cd
F value				
6-BA		84.86 ***	29.55 ***	28.62 ***
IBA		86.36 ***	23.48 ***	13.03 ***
6-BA × IBA		4.93 **	3.61 **	2.58 *

Half-strength MS medium supplemented with 3% sucrose. Values are the mean ± SD of three replicates, each with 30 explants. Means with the same letters in the same columns are not significantly different from each other at *p* < 0.05 (according to Duncan’s multiple range test). F value represented * = *p* < 0.05, ** = *p* < 0.01, and *** = *p* < 0.001.

**Table 8:** Effects of 6-BA and IBA on the frequency of shoot induction and the proliferation coefficient.

	6-BA (mg/L)		1.0	2.0	4.0	5.0	F Value
IBA (mg/L)							
0.1		Frequency of shoot induction (%)	22.22 ± 3.85 c (B)	28.89 ± 3.85 b (B)	42.22 ± 3.85 c (A)	28.89 ± 3.85 b (B)	6-BA: 84.86 ***
0.2			42.22 ± 3.85 a (B)	44.44 ± 3.85 a (B)	80.00 ± 6.67 a (A)	44.44 ± 3.85 a (B)	IBA: 86.36 ***
0.5			31.11 ± 3.85 b (B)	35.56 ± 3.85 b (B)	57.78 ± 3.85 b (A)	35.56 ± 3.85 b (B)	6-BA × IBA: 4.93 **
0.1		Net proliferation coefficient	2.70 ± 0.67 b (C)	3.00 ± 0.91 b (BC)	4.00 ± 0.82 b (A)	3.46 ± 0.52 a (AB)	6-BA: 29.55 ***
0.2			3.42 ± 0.96 a (B)	3.90 ± 1.17 a (B)	5.69 ± 1.45 a (A)	3.85 ± 0.75 a (B)	IBA: 23.48 ***
0.5			2.79 ± 0.80 b (C)	3.63 ± 0.96 ab (B)	4.35 ± 1.20 b (A)	3.69 ± 0.79 a (B)	6-BA × IBA: 3.61 **
0.1		Total proliferation coefficient	0.60 ± 1.18 b (B)	0.87 ± 1.45 b (B)	1.69 ± 2.07 b (A)	0.87 ± 1.47 b (B)	6-BA: 28.62 ***
0.2			1.44 ± 1.82 a (B)	1.73 ± 2.10 a (B)	4.56 ± 2.64 a (A)	1.73 ± 1.94 a (B)	IBA: 13.03 ***
0.5			0.87 ± 1.38 ab (B)	1.29 ± 1.84 ab (B)	2.69 ± 2.35 b (A)	1.20 ±1.78 ab (B)	6-BA × IBA: 2.58 *

Half-strength MS medium supplemented with 3% sucrose. Values are the mean ± SD of three replicates, each with 30 explants. At the 6-BA concentration, means followed by the same lowercase letter in each column are not significantly different from each other at *p* < 0.05 (according to L.S.D. test). At the same IBA concentration, means followed by the same uppercase letter (in parenthesis) in each row are not significantly different from each other at *p* < 0.05 (according to L.S.D. test). F value represented * = *p* < 0.05, ** = *p* < 0.01, and *** = *p* < 0.001.

**Table 9:** Effect of 6-BA and IAA on adventitious shoot elongation.

6-BA (mg/L)	IAA (mg/L)	Mean Length of Adventitious Shoots (cm)
0	1.0	1.38 ± 0.25 d
0	1.5	1.63 ± 0.23 bc
0	2.0	1.57 ± 0.23 c
0.1	1.0	1.47 ± 0.22 d
0.1	1.5	1.93 ± 0.19 a
0.1	2.0	1.68 ± 0.22 bc
0.2	1.0	1.42 ± 0.25 d
0.2	1.5	1.69 ± 0.24 b
0.2	2.0	1.62 ± 0.22 bc
F value		
6-BA		16.34 ***
IAA		62.42 ***
6-BA × IAA		2.97 *

Half-strength MS medium supplemented with 3% sucrose. Values are the mean ± SD of three replicates, each with 30 explants. Means with the same letters in the same columns are not significantly differentfrom each other at *p* < 0.05 (according to Duncan’s multiple range test). F value represented * = *p* < 0.05, and *** = *p* < 0.001.

**Table 10:** Effect of 6-BA and IAA on the mean length of adventitious shoots (cm).

	IAA (mg/L)	0	0.1	0.2
6-BA (mg/L)				
1.0		1.38 ± 0.25 b (A)	1.47 ± 0.22 c (A)	1.42 ± 0.25 b (A)
1.5		1.63 ± 0.23 a (B)	1.93 ± 0.19 a (A)	1.69 ± 0.24 a (B)
2.0		1.57 ± 0.23 a (B)	1.68 ± 0.22 b (A)	1.62 ± 0.22 a (AB)
F value				
6-BA: 16.34 *** IAA: 62.42 *** 6-BA × IAA: 2.97 *				

Half-strength MS medium supplemented with 3% sucrose. Values are the mean ± SD of three replicates, each with 30 explants. At the 6-BA concentration, means followed by the same lowercase letter in each column are not significantly different from each other at *p* < 0.05 (according to L.S.D. test). At the same IAA concentration, means followed by the same uppercase letter (in parenthesis) in each row are not significantly different from each other at *p* < 0.05 (according to L.S.D. test). F value represented * = *p* < 0.05, and *** = *p* < 0.001.

**Table 11:** Effect of NAA on rooting of adventitious shoot.

NAA (mg/L)	Rooting Percentage (%)	Mean number of Roots Per Adventitious Shoot	Mean Length of Roots (cm)
0	42.22 ± 3.85 c	2.47 ± 0.96 c	1.72 ± 0.55 d
0.1	82.00 ± 3.85 a	4.84 ± 1.14 a	3.91 ± 0.95 a
0.2	73.33 ± 6.67 b	4.45 ± 0.98 a	3.52 ± 0.71 b
0.5	48.89 ± 3.85 c	3.63 ± 0.90 b	3.11 ± 0.88 c

Half-strength MS medium supplemented with 3% sucrose. Values are the mean ± SD. of three replicates, each with 30 explants. Means with the same letters in the same columns are not significantly different from each other at *p* < 0.05 (according to Duncan’s multiple range test).

## Data Availability

The data presented in this study are available in the article.

## Abbreviations

MS	Murashige and Skoog
1/2 MS	Half-strength Murashige and Skoog
PPM	Plant Preservative Mixture
6-BA	6-benzyladenine
IBA	Indole-3-butyric acid
IAA	Indole-3-acetic acid
NAA	α-Naphthalene acetic acid
PGRs	Plant growth regulators
